# Supplementary material for: General lightweight framework for vision foundation model supporting multi-task and multi-center medical image analysis
Source: Nat Commun. 2025 Mar 1;16:2097. doi: 10.1038/s41467-025-57427-z (PMC11873151; doi:10.1038/s41467-025-57427-z)
Supplement: Supplementary file 1 — Supplementary Information [file 41467_2025_57427_MOESM1_ESM.pdf]

Supplementary Note 1: Data preprocessing

The preprocessing process of CT images is shown in Supplementary Fig.6. Firstly, a physician identifies the rectangular region of interest (ROI) in the original CT images and performs cropping. The rectangular ROI encloses the contour of the entire uterus. Subsequently, the ROI image is adjusted to a 224×224 square.

Supplementary Note 2: robust feature transfer networks

This study constructs two robust feature transfer (RFT) networks to achieve robust feature transfer from VFM to CNN. Based on equation (1), the representation difference between VFM and CNN for the same data is measured. By minimizing this term, the learning process of CNN to capture the representation from VFM is constrained:

$$L_2 = \left\| r_\theta (C_\theta^n (x)) - V^m (x) \right\|_2^2 \tag{1}$$

where, $(m,n) \in \partial$, ∂ is the predefined set of model layer matches, where the total number of matched pairs is denoted as *P*. $V^m(x)$ represents the intermediate feature map of the *m*th model layer in the VFM, while $C_{\theta_k}^n(x)$ represents the intermediate feature map of the *n*th model layer in the CNN model *k*. r_{θ_k} is a linear transformation parameterized by θ_k for point-wise convolutions, ensuring that the number of feature maps between the two models is consistent. Additionally, this parameter is only updated and used during the training of the target domain model, forming the target model parameters θ_k along with C_{θ_k}. x and y represent the input data and true labels, respectively.

Each convolutional layer of the CNN consists of multiple convolutional kernels. The CNN extracts data features through these convolutional kernels, with each kernel generating an intermediate feature map. After linear transformation, these intermediate feature maps are matched one by one with the intermediate feature maps generated by the transformer block of VFM, forming the feature transfer channels. We employ RFT network1 ϕ_k to calculate the weights $\omega_c^{m,n}$ for each feature transfer channel. The structure of RFT network 1 consists of *P* sets of fully connected layers, pooling layers, and a softmax layer. This network takes the features from the VFM as input and produces the output through the softmax layer. In the equation (2), $\omega_c^{m,n}$ is a learnable parameter that facilitates the matching of the *m*th layer feature from the VFM with the *n*th layer feature from the CNN model.

$$\omega_c^{m,n} = f_{\phi_k}^{m,n}(V^m(x)) \tag{2}$$

Then, we employ RFT network 2 to calculate the weights $\lambda_k^{m,n}$ for model layer matching. The structure of RFT network 2 consists of *P* sets of fully connected layers, pooling layers, and a

ReLU6 layer, forming a fully connected network. This network takes the features from the VFM as input and produces the output through the ReLU6 activation layer.

$$\lambda_k^{m,n} = g_{\phi_k}^{m,n}(V^m(x)) \tag{3}$$

where, $\lambda_k^{m,n} > 0$, and the initial value is 1.0. RFT networks ϕ_k are updated based on the change in loss of the CNN network, and the details are illustrated in Algorithm 1.

Supplementary Note 3: The construction of VFMGL robustness critical layers

By combining equations (1) and (2), the feature channel transfer loss (4) can be obtained.

$$L_{\text{wfm}}^{m,n}(\theta_k \mid x, \omega^{m,n}) = \frac{1}{HW}\sum\nolimits_c \omega_c^{m,n} \sum\nolimits_{i,j} (r_{\theta_k}(C_{\theta_k}^n(x))_{c,i,j} - V^m(x)_{c,i,j})^2 \tag{4}$$

where, $H \times W$ represents the size of the output feature maps from $(r_{\theta_k}(C_{\theta_k}^n(x))$ and $V^m(x)$.

In combination with formula (3), knowledge transfer total loss as follows:

$$L_{\text{wfm}}^{m,n}(\theta_k \mid x, \phi_k) = \sum\nolimits_{(m,n\in\partial)} \lambda_k^{m,n} L_{\text{wfm}}^{m,n}(\theta_k \mid x, \omega^{m,n}) \tag{5}$$

VFMGL's total training loss in Stage I is shown in Equation (6), where L_{org} represents the local model's standard cross-entropy loss(the segmentation task employs the dice loss). The VFMGL training process is visible in Algorithm 1.

$$L_{\text{stageI}}(\theta_k \mid x, y, \phi_k) = L_{\text{wfm}}^{m,n}(\theta_k \mid x, \phi_k) + L_{\text{org}}(\theta_k \mid x, y) \tag{6}$$

Algorithm 1

Input: the number of communication rounds n, the number of centers K, the number of training rounds for the local model E, the number of internal cycles of knowledge transfer N, and the learning rate α, the number of randomly sampled data batches B, Data batch selection threshold TV.

Output: Local model parameters $\theta_1 \dots \theta_k \dots \theta_K$.

```
Initialize the model parameters θ_k , load the VFM pre-trained model parameters
For i=1 to n do
    For k=1 to K do
        For e=1 to E do
            For j=1 to N do
                θ_k ← θ_k − a∇_θ L_wfm(θ_k | x, φ_k)
                θ_k ← θ_k − a∇_θ L_org(θ_k | x, y)
                φ_k ← φ_k − a∇_φ L_org(θ_k | x, y)
            Return θ_k
            Compute L
            If i=1:
                Medical center sends θ_k to the server
                Aggregate shared model θ_shared ← (Σ_{k=1}^{K} θ_k) / K
                Server sends θ_shared to each medical center
            Else:
                For b in B do
                    Batch_data=random_select(data)
                    Compute KL with batch data
                    If KL/L<TV:
                        θ_k ← θ_k − a∇_θ L_stageII(θ_k | x, y)
                Return θ_k
                Medical center sends θ_k to the server for aggregation of the shared model
End
```

Supplementary Note 4: DDBL

In a multi-center setting with distributional heterogeneity, it is possible that some data possess better commonality. Based on this assumption, we have constructed a straightforward data deduction framework to identify local data that better aligns with the global data distribution for local model training. In FL, each center has access to both the globally distributed model parameters sent by the server and the locally trained model parameters. By randomly sampling a batch and feeding it into both the shared and local models simultaneously, we input the feature maps from the last model layer (before the Avgpool layer) of both models into the classification layer of the shared model, generating two sets of predicted probability distributions(For segmentation models, the feature maps of the last encoding layer of the model are inputted into the decoding layer of the shared model.). We then compute the Kullback-Leibler(KL) divergence based on equation (7). Simultaneously, by utilizing the predicted probability distribution of the local model and the true label distribution, we calculate the distribution discrepancy based on equation (8).

$$KL = -\sum_i Q_1(i)\log(\frac{Q_1(i)}{Q_2(i)}) \tag{7}$$

$$L = -\sum_i P(i)\log(Q_3(i)) \tag{8}$$

In the above equations, Q_1 represents the predicted probability distribution output by the shared model based on the local model representation, Q_2 represents the predicted probability distribution output by the shared model based on its own representation, and P represents the true label distribution. i denotes the index of the category. Q_3 represents the probability distribution of predictions based on the outputs of the local model's representations.

We use KL/L to measure the heterogeneity of batch data. With the iteration of local model training, for data with small heterogeneity, both the L value and KL value will continuously decrease. On the other hand, for data with strong heterogeneity, the KL value will be challenging to reduce, while the L value will decrease rapidly. Therefore, we set a threshold (TV, default value 4) to filter out batches of data with significant heterogeneity. The details are illustrated in Algorithm 1.

Supplementary Note 5: The training strategy via DDBL

This study constructs the knowledge distillation loss function for the second stage of VFMGL based on KL divergence (Equation (9)). After freezing the robustness critical layer obtained in Stage I, the remaining model layers of the local model are constrained to acquire multi-center knowledge from the shared model.

$$L_{\mathrm{KL}}(\theta_k \mid z_1, z_2) = \sum_i \left(\log\left(\frac{\exp(z_1(i)/temp)/\sum_j \exp(z_1(j)/temp)}{\exp(z_2(i)/temp)/\sum_j \exp(z_2(j)/temp)}\right) \bullet \frac{\exp(z_1(i)/temp)}{\sum_j \exp(z_1(j)/temp)}\right) \quad (9)$$

Where, z_1 and z_2 are probability distributions calculated by the shared model classification head based on the representations generated by the local model and the shared model for the same sample, respectively. $z_1(i)$ and $z_2(i)$ represent the probabilities of the ith sample for each distribution. Here, i represents a category in the predicted distribution, and j represents the index of all categories. Hyper-parameter *temp* is used to control the smoothness of the probability distribution.

Combining Equation (9) with the standard cross-entropy L_{org}, the total loss function for Stage II is given by Equation (10).

$$L_{\mathrm{stageII}}(\theta_k \mid x, y) = (1-\beta) \bullet L_{\mathrm{KL}}(\theta_k \mid z_1, z_2) + \beta L_{\mathrm{org}}(\theta_k \mid x, y) \quad (10)$$

Where β is a hyper-parameter between 0 and 1, used to balance the relative importance of the two loss terms, with a default value of 0.5.

Supplementary Note 6: Adaptive knowledge and common knowledge of VFMGL

The analysis of adaptive knowledge and common knowledge in VFMGL involves two main steps. Initially, local models from each center extract features from the test and train datasets of all centers. Following this, the extracted features undergo a round of Max-Relevance and Min-Redundancy (mRMR) feature selection to identify 256 deep learning (DL) features. Subsequently, the DL features are categorized into two groups based on NMI and MI to eliminate differences between categories. A correlation analysis is then performed on the DL features of NMI and MI, and a correlation heat map is constructed. From this analysis, features exhibiting the highest correlation within the same center and the lowest correlation across different data centers are identified as adaptive knowledge. Conversely, features displaying the highest correlation across different data centers are considered common knowledge.

Supplementary Note 7: Classifier

For classification tasks, VFMGL is not limited to specific classification layers or classifiers. For small-sample datasets, to enhance the utilization of model features, each data center can utilize the convolutional kernels of its own local model as feature extractors. Multiple feature maps are extracted from the local image data of each patient in the local dataset. Subsequently, the average value of each feature map is computed to obtain a set of unified DL features. Finally, sparse Bayesian extreme learning machines [1] are used to construct the prediction labels (Supplementary Fig.7).

Supplementary Table 1. Comparison of the performance of six methods in use case 1

Center	Methods	AUC	Sensitivity	Specificity	Accuracy	PPV	NPV	F1
A	FedAvg	0.676	0.461(111/241)	0.794(27/34)	0.502(138/275)	0.941(111/118)	0.172(27/157)	0.619
	FedProx	0.702	0.448(108/241)	0.824(28/34)	0.495(136/275)	0.947(108/114)	0.174(28/161)	0.608
	HarmoFL	0.719	0.685(165/241)	0.647(22/34)	0.680(187/275)	0.932(165/177)	0.224(22/98)	0.790
	MetaFed	0.732	0.705(170/241)	0.647(22/34)	0.698(192/275)	0.934(170/182)	0.237(22/93)	0.804
	Virchow	0.716	0.622(150/241)	0.706(24/34)	0.633(174/275)	0.938(150/160)	0.209(24/115)	0.748
	VFMGL	0.798	0.884(213/241)	0.500(17/34)	0.836(230/275)	0.926(213/230)	0.378(17/45)	0.905
B	FedAvg	0.710	0.696(16/23)	0.500(3/6)	0.655(19/29)	0.842(16/19)	0.300(3/10)	0.762
	FedProx	0.754	0.739(17/23)	0.667(4/6)	0.724(21/29)	0.895(17/19)	0.400(4/10)	0.810
	HarmoFL	0.790	0.739(17/23)	0.667(4/6)	0.724(21/29)	0.895(17/19)	0.400(4/10)	0.810
	MetaFed	0.775	0.609(14/23)	0.667(4/6)	0.621(18/29)	0.875(14/16)	0.308(4/13)	0.718
	Virchow	0.804	0.826(19/23)	0.667(4/6)	0.793(23/29)	0.905(19/21)	0.500(4/8)	0.864
	VFMGL	0.833	0.565(13/23)	0.833(5/6)	0.621(18/29)	0.929(13/14)	0.333(5/15)	0.703
C	FedAvg	0.643	0.571(8/14)	1.000(1/1)	0.600(9/15)	1.000(8/8)	0.143(1/7)	0.727
	FedProx	0.714	0.714(10/14)	0.000(0/1)	0.667(10/15)	0.909(10/11)	0.000(0/4)	0.800
	HarmoFL	0.786	0.929(13/14)	0.000(0/1)	0.867(13/15)	0.929(13/14)	0.000(0/1)	0.929
	MetaFed	0.786	0.786(11/14)	1.000(1/1)	0.800(12/15)	1.000(11/11)	0.250(1/4)	0.880
	Virchow	0.786	0.857(12/14)	0.000(0/1)	0.800(12/15)	0.923(12/13)	0.000(0/2)	0.889
	VFMGL	0.857	0.857(12/14)	0.000(0/1)	0.800(12/15)	0.923(12/13)	0.000(0/2)	0.889
D	FedAvg	0.765	0.882(97/110)	0.500(4/8)	0.856(101/118)	0.960(97/101)	0.235(4/17)	0.919
	FedProx	0.752	0.500(55/110)	1.000(8/8)	0.534(63/118)	1.000(55/55)	0.127(8/63)	0.667
	HarmoFL	0.805	0.582(64/110)	0.875(7/8)	0.602(71/118)	0.985(64/65)	0.132(7/53)	0.732
	MetaFed	0.807	0.836(92/110)	0.500(4/8)	0.814(96/118)	0.958(92/96)	0.182(4/22)	0.893
	Virchow	0.823	0.636(70/110)	0.875(7/8)	0.653(77/118)	0.986(70/71)	0.149(7/47)	0.773
	VFMGL	0.848	0.745(82/110)	0.875(7/8)	0.754(89/118)	0.988(82/83)	0.200(7/35)	0.849

Notes: VFMGL, Vision Foundation Model General Lightweight; AUC, Area Under the Curve; PPV, Positive Predictive Value; NPV, Negative Predictive Value. Source data are provided as a Source Data file.

Supplementary Table 2. Comparison of the performance of six methods in use case 2

Center	Methods	AUC	Sensitivity	Specificity	Accuracy	PPV	NPV	F1
A	FedAvg	0.9358	0.8484 (5043/5944)	0.9561 (5683/5944)	0.9023 (10726/11888)	0.9508 (5043/5304)	0.8632 (5683/6584)	0.8967
	FedProx	0.9875	0.9263 (5506/5944)	0.9739 (5789/5944)	0.9501 (11295/11888)	0.9726 (5506/5661)	0.9297 (5789/6227)	0.9489
	HarmoFL	0.9863	0.9127 (5425/5944)	0.9754 (5798/5944)	0.9441 (11223/11888)	0.9738 (5425/5571)	0.9178 (5798/6317)	0.9423
	MetaFed	0.9874	0.9268 (5509/5944)	0.9798 (5824/5944)	0.9533 (11333/11888)	0.9787 (5509/5629)	0.9305 (5824/6259)	0.9520
	Virchow	0.9831	0.9633 (5726/5944)	0.9680 (5754/5944)	0.9657 (11480/11888)	0.9679 (5726/5916)	0.9635 (5754/5972)	0.9656
	VFMGL	0.9992	0.9882 (5874/5944)	0.9896 (5882/5944)	0.9889 (11756/11888)	0.9896 (5874/5936)	0.9882 (5882/5952)	0.9889
B	FedAvg	0.8420	0.7946 (2773/3490)	0.8479 (2960/3491)	0.8212 (5733/6981)	0.8393 (2773/3304)	0.8050 (2960/3677)	0.8163
	FedProx	0.9711	0.9060 (3162/3490)	0.9215 (3217/3491)	0.9138 (6379/6981)	0.9203 (3162/3436)	0.9075 (3217/3545)	0.9131
	HarmoFL	0.9658	0.8837 (3084/3490)	0.9184 (3206/3491)	0.9010 (6290/6981)	0.9154 (3084/3369)	0.8876 (3206/3612)	0.8993
	MetaFed	0.9776	0.9095 (3174/3490)	0.9433 (3293/3491)	0.9264 (6467/6981)	0.9413 (3174/3372)	0.9124 (3293/3609)	0.9251
	Virchow	0.9795	0.8123 (2835/3490)	0.9989 (3487/3491)	0.9056 (6322/6981)	0.9986 (2835/2839)	0.8419 (3487/4142)	0.8959
	VFMGL	0.9973	0.9481 (3309/3490)	0.9974 (3482/3491)	0.9728 (6791/6981)	0.9973 (3309/3318)	0.9506 (3482/3663)	0.9721
C	FedAvg	0.9713	0.9155 (7787/8506)	0.9509 (8087/8505)	0.9332 (15874/17011)	0.9491 (7787/8205)	0.9184 (8087/8806)	0.9320
	FedProx	0.9892	0.9474 (8059/8506)	0.9714 (8262/8505)	0.9594 (16321/17011)	0.9707 (8059/8302)	0.9487 (8262/8709)	0.9589
	HarmoFL	0.9829	0.9319 (7927/8506)	0.9566 (8136/8505)	0.9443 (16063/17011)	0.9555 (7927/8296)	0.9336 (8136/8715)	0.9436
	MetaFed	0.9886	0.9452 (8040/8506)	0.9688 (8240/8505)	0.9570 (16280/17011)	0.9681 (8040/8305)	0.9465 (8240/8706)	0.9565
	Virchow	0.9670	0.8594 (7310/8506)	0.9805 (8339/8505)	0.9199 (15649/17011)	0.9778 (7310/7476)	0.8746 (8339/9535)	0.9148
	VFMGL	0.9995	0.9911 (8430/8506)	0.9915 (8433/8505)	0.9913 (16863/17011)	0.9915 (8430/8502)	0.9911 (8433/8509)	0.9913
D	FedAvg	0.9763	0.9067 (11772/12984)	0.9546 (12394/12984)	0.9306 (24166/25968)	0.9523 (11772/12362)	0.9109 (12394/13606)	0.9289
	FedProx	0.9925	0.9478 (12306/12984)	0.9784 (12704/12984)	0.9631 (25010/25968)	0.9778 (12306/12586)	0.9493 (12704/13382)	0.9626
	HarmoFL	0.9856	0.9216 (11966/12984)	0.9718 (12618/12984)	0.9467 (24584/25968)	0.9703 (11966/12332)	0.9253 (12618/13636)	0.9453
	MetaFed	0.9927	0.9477 (12305/12984)	0.9823 (12754/12984)	0.9650 (25059/25968)	0.9817 (12305/12535)	0.9495 (12754/13433)	0.9644
	Virchow	0.9736	0.6842 (8883/12984)	0.9992 (12973/12984)	0.8417 (21856/25968)	0.9988 (8883/8894)	0.7598 (12973/17074)	0.8120
	VFMGL	0.9977	0.9427 (12240/12984)	0.9988 (12969/12984)	0.9708 (25209/25968)	0.9988 (12240/12255)	0.9457 (12969/13713)	0.9699
E	FedAvg	0.9827	0.9439 (13849/14672)	0.9567 (14038/14673)	0.9503 (27887/29345)	0.9562 (13849/14484)	0.9446 (14038/14861)	0.9500
	FedProx	0.9946	0.9600 (14085/14672)	0.9808 (14392/14673)	0.9704 (28477/29345)	0.9804 (14085/14366)	0.9608 (14392/14979)	0.9701
	HarmoFL	0.9912	0.9624 (14121/14672)	0.9554 (14019/14673)	0.9589 (28140/29345)	0.9557 (14121/14775)	0.9622 (14019/14570)	0.9590
	MetaFed	0.9947	0.9625 (14122/14672)	0.9761 (14322/14673)	0.9693 (28444/29345)	0.9757 (14122/14473)	0.9630 (14322/14872)	0.9691
	Virchow	0.9589	0.8676 (12729/14672)	0.9826 (14417/14673)	0.9251 (27146/29345)	0.9803 (12729/12985)	0.8812 (14417/16360)	0.9205
	VFMGL	0.9993	0.9845 (14445/14672)	0.9922 (14559/14673)	0.9884 (29004/29345)	0.9922 (14445/14559)	0.9846 (14559/14786)	0.9883

Notes: VFMGL, Vision Foundation Model General Lightweight; AUC, Area Under the Curve; PPV, Positive Predictive Value; NPV, Negative Predictive Value. Source data are provided as a Source Data file.

Supplementary Table 3. Comparison of the performance of six methods in use case 3

Center	Methods	Dice	ASSD	Sensitivity	Specificity	PPV	IOU	F1
A	FedAvg	0.5183	55.6626	0.7629	0.7337	0.1240	0.1186	0.2133
	FedProx	0.8268	23.4177	0.6776	0.9894	0.7460	0.5468	0.7102
	HarmoFL	0.9253	6.7873	0.8624	0.9951	0.8711	0.7481	0.8667
	MetaFed	0.7531	23.1151	0.5629	0.9897	0.7364	0.4758	0.6381
	MedSAM	0.9163	5.3587	0.7665	0.9989	0.9682	0.7399	0.8556
	VFMGL	0.9340	5.6570	0.8981	0.9953	0.8685	0.7872	0.8831
B	FedAvg	0.5034	52.5426	0.8354	0.7513	0.0840	0.0827	0.1527
	FedProx	0.8879	15.7715	0.8247	0.9929	0.7207	0.6001	0.7692
	HarmoFL	0.9390	7.5250	0.9570	0.9935	0.7534	0.7162	0.8431
	MetaFed	0.8995	8.4520	0.8583	0.9948	0.7854	0.6682	0.8202
	MedSAM	0.8810	4.8425	0.6994	0.9993	0.9614	0.6651	0.8098
	VFMGL	0.9328	5.0626	0.9337	0.9956	0.8196	0.7659	0.8729
C	FedAvg	0.7868	25.7947	0.9805	0.9543	0.4144	0.4060	0.5826
	FedProx	0.9037	6.6428	0.8132	0.9968	0.8836	0.7172	0.8469
	HarmoFL	0.9525	4.5665	0.9597	0.9939	0.8232	0.7895	0.8862
	MetaFed	0.8913	9.2436	0.8297	0.9912	0.7496	0.6494	0.7876
	MedSAM	0.8965	5.7118	0.7270	0.9988	0.9594	0.6929	0.8272
	VFMGL	0.9620	2.8192	0.9539	0.9973	0.9076	0.8672	0.9302
D	FedAvg	0.6957	45.0055	0.7222	0.9320	0.2818	0.2584	0.4054
	FedProx	0.8536	18.7689	0.7450	0.9919	0.7242	0.5928	0.7345
	HarmoFL	0.9084	13.8240	0.9455	0.9892	0.7334	0.6855	0.8261
	MetaFed	0.8716	15.8724	0.7776	0.9880	0.6874	0.5869	0.7297
	MedSAM	0.9098	4.2473	0.7565	0.9995	0.9840	0.7416	0.8554
	VFMGL	0.9191	9.4369	0.8434	0.9966	0.8370	0.7465	0.8402
E	FedAvg	0.7557	44.8552	0.8460	0.9364	0.3739	0.3555	0.5186
	FedProx	0.9018	14.1736	0.8811	0.9896	0.7645	0.6925	0.8187
	HarmoFL	0.9345	6.8490	0.8794	0.9948	0.8569	0.7654	0.8680
	MetaFed	0.8151	19.1216	0.6407	0.9884	0.7034	0.5449	0.6706
	MedSAM	0.9129	4.9324	0.7698	0.9985	0.9661	0.7403	0.8569
	VFMGL	0.9383	6.0202	0.9296	0.9946	0.8501	0.8028	0.8881
F	FedAvg	0.5078	53.7082	0.7025	0.7794	0.0880	0.0856	0.1564
	FedProx	0.7754	27.9849	0.7216	0.9880	0.6303	0.5371	0.6729
	HarmoFL	0.9167	12.9202	0.8876	0.9897	0.7471	0.7012	0.8113
	MetaFed	0.8268	18.1592	0.6701	0.9874	0.6345	0.5282	0.6518
	MedSAM	0.9076	4.3989	0.7476	0.9994	0.9644	0.7154	0.8423
	VFMGL	0.9080	6.8139	0.8981	0.9942	0.7973	0.7445	0.8447

Notes: VFMGL, Vision Foundation Model General Lightweight; ASSD, Average Symmetric Surface Distance; PPV, Positive Predictive Value; IOU: Intersection Over Union. Source data are provided as a Source Data file.

Supplementary Table 4. Comparison of the performance of six methods in use case 4

Center	Methods	Dice	ASSD	Sensitivity	Specificity	PPV	IOU	F1
A	FedAvg	0.4493	5.5991	0.3041	0.5673	0.2572	0.1620	0.2787
	FedProx	0.7175	4.0955	0.6210	0.9322	0.7564	0.4858	0.6820
	HarmoFL	0.7126	4.1270	0.5655	0.9516	0.7929	0.4597	0.6602
	MetaFed	0.7066	4.2787	0.5823	0.9420	0.7715	0.4641	0.6637
	MedSAM	0.7703	1.8837	0.6338	0.8752	0.7653	0.5256	0.6934
	VFMGL	0.7509	3.6429	0.5774	0.9606	0.8257	0.4918	0.6796
B	FedAvg	0.4740	5.3538	0.3170	0.6262	0.2570	0.1647	0.2839
	FedProx	0.7140	4.3436	0.6542	0.9075	0.6687	0.4589	0.6614
	HarmoFL	0.7165	4.3308	0.5974	0.9358	0.7194	0.4509	0.6527
	MetaFed	0.6894	4.4940	0.6227	0.9056	0.6627	0.4413	0.6421
	MedSAM	0.7666	2.4434	0.6379	0.8743	0.6937	0.5073	0.6646
	VFMGL	0.7658	4.1422	0.6245	0.9347	0.7240	0.4731	0.6706
C	FedAvg	0.4336	10.2695	0.2468	0.5464	0.1723	0.1117	0.2029
	FedProx	0.7643	8.6537	0.7056	0.9073	0.6443	0.4772	0.6736
	HarmoFL	0.7696	8.4898	0.6516	0.9345	0.6886	0.4711	0.6696
	MetaFed	0.7199	8.7500	0.6851	0.8927	0.6213	0.4703	0.6516
	MedSAM	0.8219	2.5373	0.6901	0.9297	0.7839	0.5936	0.7340
	VFMGL	0.7735	8.2582	0.6203	0.9646	0.7653	0.4823	0.6852
D	FedAvg	0.4663	5.8823	0.3054	0.5668	0.2514	0.1587	0.2758
	FedProx	0.7080	4.3680	0.5639	0.9435	0.7748	0.4704	0.6527
	HarmoFL	0.6983	4.2005	0.5113	0.9616	0.8142	0.4459	0.6281
	MetaFed	0.6723	4.5729	0.5402	0.9410	0.7670	0.4466	0.6339
	MedSAM	0.7911	2.1497	0.6590	0.9024	0.7635	0.5534	0.7074
	VFMGL	0.7410	3.6780	0.5059	0.9681	0.8440	0.4471	0.6326
E	FedAvg	0.5288	1.0811	0.3998	0.6715	0.3341	0.2216	0.3640
	FedProx	0.6645	1.2186	0.5603	0.8924	0.6787	0.4406	0.6138
	HarmoFL	0.6901	1.1861	0.5380	0.9349	0.7690	0.4616	0.6331
	MetaFed	0.7016	1.1509	0.5698	0.9178	0.7382	0.4713	0.6432
	MedSAM	0.6591	1.2267	0.5590	0.7690	0.5127	0.3648	0.5349
	VFMGL	0.7568	1.0707	0.6386	0.8806	0.6821	0.4914	0.6596
F	FedAvg	0.4406	10.1532	0.3125	0.5589	0.1315	0.1010	0.1851
	FedProx	0.6677	7.3864	0.6236	0.8654	0.4064	0.2877	0.4921
	HarmoFL	0.6961	6.8815	0.6171	0.9039	0.4793	0.3260	0.5395
	MetaFed	0.6834	6.9784	0.6559	0.8965	0.4763	0.3391	0.5519
	MedSAM	0.8157	1.2948	0.6114	0.9693	0.7693	0.5180	0.6813
	VFMGL	0.7899	4.1967	0.6218	0.9729	0.7339	0.4772	0.6732

Notes: VFMGL, Vision Foundation Model General Lightweight; ASSD, Average Symmetric Surface Distance; PPV, Positive Predictive Value; IOU: Intersection Over Union. Source data are provided as a Source Data file.

Supplementary Table 5. Distribution of the use case 1 Test-Set data based on age grouping

	Group	Center A		Center B		Center C		Center D	
		NMI	MI	NMI	MI	NMI	MI	NMI	MI
Test-Set	Group1(Age<=54.7)	29	122	5	10	1	4	8	55
	Group2(Age>54.7)	5	119	1	13	0	10	0	55

Notes: MI, Myometrial Invasion; NMI, Non Myometrial Invasion.

Supplementary Table 6. Multiple permutation distribution for use case 1

Distribution	Random Seed	partition ratio	Set	Center A		Center B		Center C		Center D	
				NMI	MI	NMI	MI	NMI	MI	NMI	MI
Initial	42	0.60	TrainSet	49	361	8	34	1	21	11	165
			TestSet	34	241	6	23	1	14	8	110
1	50	0.50	TrainSet	41	301	7	28	1	17	9	137
			TestSet	42	301	7	29	1	18	10	138
2	100	0.55	TrainSet	45	331	7	31	1	19	10	151
			TestSet	38	271	7	26	1	16	9	124
3	200	0.65	TrainSet	53	391	9	37	1	22	12	178
			TestSet	30	211	5	20	1	13	7	97
4	500	0.70	TrainSet	58	421	9	39	1	24	13	192
			TestSet	25	181	5	18	1	11	6	83
5	1000	0.80	TrainSet	66	481	11	45	1	28	15	220
			TestSet	17	121	3	12	1	7	4	55

Notes: MI, Myometrial Invasion; NMI, Non Myometrial Invasion.

Supplementary Table 7. Robustness critical layers for local model of each medical center in use case 1

Center	layer0		layer1		layer2		layer3		layer4	
A	0.87267068		1.68551706	√	1.66083648	√	1.49487238	√	1.18845993	
B	0.44240588		2.43922249	√	0.66256395	√	1.36607569	√	0.61106268	
C	1.77630597	√	1.81546049	√	0.89839248		0.69220563		1.06292467	√
D	0.87161931	√	1.28099127	√	0.83994865	√	0.79902224		0.67803999	

In use case 1, we employ the DINOv2 model as the source model and ResNet18 as the expert model for clinical diagnosis, transferring the first convolutional layer and the subsequent four blocks of ResNet18 as the destination. The models layers marked in the table are identified as critical for robustness.

Supplementary Table 8. Data distribution of use case 1-4

EC-MI Classification(use case 1)

	A	B	C	D	external center E	external center F
Train Set	410	42	22	176	-	-
Test Set	275	29	15	118	63	117

breast cancer histopathological image classification(use case2)

	A	B	C	D	E
Train Set	38039	22339	54435	83096	93902
Val Set	9509	5584	13608	20774	23475
Test Set	11888	6981	17011	25968	29345

prostate MRI segmentation(use case 3)

	A	B	C	D	E	F
Train Set	167	100	299	263	245	112
Val Set	41	25	75	65	62	28
Test Set	53	32	94	83	77	35

histological cell nucleus segmentation(use case 4)

	A	B	C	D	E	F
Train Set	47	31	44	46	24	26
Val Set	11	8	11	11	6	7
Test Set	22	22	31	26	14	17

Notes: EC, Endometrial Cancer; MI, Myometrial Invasion. MRI, Magnetic Resonance Imaging. Source data are provided as a Source Data file.

Supplementary Table 9. Multiple permutation distribution for use case 2

Distribution	Random Seed	partition ratio	Set	Center A	B	C	D	E
	initial		Train Set	38039	22339	54435	83096	93902
			Val Set	9509	5584	13608	20774	23475
			Test Set	11888	6981	17011	25968	29345
1	50	0.50	Train Set	23775	13962	34023	51936	58690
			Val Set	5943	3490	8505	12984	14672
			Test Set	29718	17452	42526	64918	73360
2	100	0.55	Train Set	26152	15359	37424	57130	64559
			Val Set	6538	3839	9356	14282	16139
			Test Set	26746	15706	38274	58426	66024
3	200	0.65	Train Set	30908	18151	44229	67517	76296
			Val Set	7726	4537	11057	16879	19074
			Test Set	20802	12216	29768	45442	51352
4	500	0.70	Train Set	33285	19548	47631	72711	82165
			Val Set	8321	4886	11907	18177	20541
			Test Set	17830	10470	25516	38950	44016
5	1000	0.80	Train Set	38040	22340	54436	83098	93903
			Val Set	9510	5584	13608	20774	23475
			Test Set	11886	6980	17010	25966	29344

We shuffled the dataset using a random seed and then split it into training and test sets according to the specified ratio. Then, 20% of the training set was extracted to be used as the validation set. Source data are provided as a Source Data file.

Supplementary Table 10. Multiple permutation distribution for use case 3

Distribution	Random Seed	partition ratio	Set	Center A	Center B	Center C	Center D	Center E	Center F
	initial		Train Set	167	100	299	263	245	112
			Val Set	41	25	75	65	62	28
			Test Set	53	32	94	83	77	35
1	50	0.50	Train Set	104	62	187	164	153	70
			Val Set	26	16	47	41	39	17
			Test Set	131	79	234	206	192	88
2	100	0.55	Train Set	114	69	205	180	168	77
			Val Set	29	17	52	46	43	19
			Test Set	118	71	211	185	173	79
3	200	0.65	Train Set	135	81	243	213	199	91
			Val Set	34	21	61	54	50	22
			Test Set	92	55	164	144	135	62
4	500	0.70	Train Set	146	87	262	230	215	98
			Val Set	36	22	65	57	53	24
			Test Set	79	48	141	124	116	53
5	1000	0.80	Train Set	167	100	299	263	245	112
			Val Set	41	25	75	65	62	28
			Test Set	53	32	94	83	77	35

We shuffled the dataset using a random seed and then split it into training and test sets according to the specified ratio. Then, 20% of the training set was extracted to be used as the validation set. Source data are provided as a Source Data file.

Supplementary Table 11. Multiple permutation distribution for use case 4

Distribution	Random Seed	partition ratio	Set	Center					
				A	B	C	D	E	F
	initial		Train Set	47	31	44	46	24	26
			Val Set	11	8	11	11	6	7
			Test Set	22	22	31	26	14	17
1	50	0.50	Train Set	32	24	34	32	17	20
			Val Set	8	6	9	9	5	5
			Test Set	40	31	43	42	22	25
2	100	0.55	Train Set	35	26	37	36	19	21
			Val Set	9	7	10	9	5	6
			Test Set	36	28	39	38	20	23
3	200	0.65	Train Set	41	31	44	42	22	25
			Val Set	11	8	11	11	6	7
			Test Set	28	22	31	30	16	18
4	500	0.70	Train Set	44	33	48	46	24	28
			Val Set	12	9	12	12	6	7
			Test Set	24	19	26	25	14	15
5	1000	0.80	Train Set	51	38	54	52	28	32
			Val Set	13	10	14	14	7	8
			Test Set	16	13	18	17	9	10

We shuffled the dataset using a random seed and then split it into training and test sets according to the specified ratio. Then, 20% of the training set was extracted to be used as the validation set. Source data are provided as a Source Data file.

Supplementary Table 12. Performance of VFMGL under various data distributions in use case 2

Center	Methods	AUC	Sensitivity	Specificity	Accuracy	PPV	NPV	F1
A	initial	0.9992	0.9882 (5874/5944)	0.9896 (5882/5944)	0.9889 (11756/11888)	0.9896 (5874/5936)	0.9882 (5882/5952)	0.9889
	1	0.9989	0.9896 (14704/14859)	0.9860 (14651/14859)	0.9878 (29355/29718)	0.9861 (14704/14912)	0.9895 (14651/14806)	0.9878
	2	0.9988	0.9765 (13059/13373)	0.9951 (13308/13373)	0.9858 (26367/26746)	0.9950 (13059/13124)	0.9769 (13308/13622)	0.9857
	3	0.9991	0.9863 (10259/10401)	0.9894 (10291/10401)	0.9879 (20550/20802)	0.9894 (10259/10369)	0.9864 (10291/10433)	0.9879
	4	0.9991	0.9874 (8803/8915)	0.9920 (8844/8915)	0.9897 (17647/17830)	0.9920 (8803/8874)	0.9875 (8844/8956)	0.9897
	5	0.9990	0.9837 (5846/5943)	0.9909 (5889/5943)	0.9873 (11735/11886)	0.9908 (5846/5900)	0.9838 (5889/5986)	0.9872
B	initial	0.9973	0.9481 (3309/3490)	0.9974 (3482/3491)	0.9728 (6791/6981)	0.9973 (3309/3318)	0.9506 (3482/3663)	0.9721
	1	0.9977	0.9589 (8367/8726)	0.9936 (8670/8726)	0.9762 (17037/17452)	0.9934 (8367/8423)	0.9602 (8670/9029)	0.9758
	2	0.9978	0.9826 (7716/7853)	0.9808 (7702/7853)	0.9817 (15418/15706)	0.9808 (7716/7867)	0.9825 (7702/7839)	0.9817
	3	0.9980	0.9751 (5956/6108)	0.9903 (6049/6108)	0.9827 (12005/12216)	0.9902 (5956/6015)	0.9755 (6049/6201)	0.9826
	4	0.9981	0.9866 (5165/5235)	0.9811 (5136/5235)	0.9839 (10301/10470)	0.9812 (5165/5264)	0.9866 (5136/5206)	0.9839
	5	0.9977	0.9731 (3396/3490)	0.9911 (3459/3490)	0.9821 (6855/6980)	0.9910 (3396/3427)	0.9735 (3459/3553)	0.9819
C	initial	0.9995	0.9911 (8430/8506)	0.9915 (8433/8505)	0.9913 (16863/17011)	0.9915 (8430/8502)	0.9911 (8433/8509)	0.9913
	1	0.9985	0.9856 (20956/21263)	0.9843 (20929/21263)	0.9849 (41885/42526)	0.9843 (20956/21290)	0.9855 (20929/21236)	0.9849
	2	0.9989	0.9718 (18598/19137)	0.9948 (19037/19137)	0.9833 (37635/38274)	0.9947 (18598/18698)	0.9725 (19037/19576)	0.9831
	3	0.9989	0.9765 (14534/14884)	0.9942 (14797/14884)	0.9853 (29331/29768)	0.9940 (14534/14621)	0.9769 (14797/15147)	0.9852
	4	0.9985	0.9911 (12644/12758)	0.9676 (12345/12758)	0.9793 (24989/25516)	0.9684 (12644/13057)	0.9908 (12345/12459)	0.9796
	5	0.9987	0.9780 (8318/8505)	0.9937 (8451/8505)	0.9858 (16769/17010)	0.9935 (8318/8372)	0.9784 (8451/8638)	0.9857
D	initial	0.9977	0.9427 (12240/12984)	0.9988 (12969/12984)	0.9708 (25209/25968)	0.9988 (12240/12255)	0.9457 (12969/13713)	0.9699
	1	0.9957	0.9607 (31183/32459)	0.9819 (31870/32459)	0.9713 (63053/64918)	0.9815 (31183/31772)	0.9615 (31870/33146)	0.9710
	2	0.9968	0.9516 (27800/29213)	0.9940 (29039/29213)	0.9728 (56839/58426)	0.9938 (27800/27974)	0.9536 (29039/30452)	0.9722
	3	0.9970	0.9682 (21999/22721)	0.9883 (22456/22721)	0.9783 (44455/45442)	0.9881 (21999/22264)	0.9688 (22456/23178)	0.9781
	4	0.9956	0.9625 (18744/19475)	0.9820 (19124/19475)	0.9722 (37868/38950)	0.9816 (18744/19095)	0.9632 (19124/19855)	0.9719
	5	0.9967	0.9670 (12554/12983)	0.9879 (12826/12983)	0.9774 (25380/25966)	0.9876 (12554/12711)	0.9676 (12826/13255)	0.9772
E	initial	0.9993	0.9845 (14445/14672)	0.9922 (14559/14673)	0.9884 (29004/29345)	0.9922 (14445/14559)	0.9846 (14559/14786)	0.9883
	1	0.9986	0.9853 (36141/36680)	0.9849 (36126/36680)	0.9851 (72267/73360)	0.9849 (36141/36695)	0.9853 (36126/36665)	0.9851
	2	0.9987	0.9915 (32730/33012)	0.9798 (32345/33012)	0.9856 (65075/66024)	0.9800 (32730/33397)	0.9914 (32345/32627)	0.9857
	3	0.9989	0.9837 (25257/25676)	0.9921 (25472/25676)	0.9879 (50729/51352)	0.9920 (25257/25461)	0.9838 (25472/25891)	0.9878
	4	0.9989	0.9913 (21816/22008)	0.9852 (21683/22008)	0.9883 (43499/44016)	0.9853 (21816/22141)	0.9912 (21683/21875)	0.9883
	5	0.9990	0.9913 (14544/14672)	0.9851 (14453/14672)	0.9882 (28997/29344)	0.9852 (14544/14763)	0.9912 (14453/14581)	0.9882

Notes: AUC, Area Under the Curve; PPV, Positive Predictive Value; NPV, Negative Predictive Value. Source data are provided as a Source Data file.

Supplementary Table 13. Performance of VFMGL under various data distributions in use case 3

Center	Methods	Dice	ASSD	Sensitivity	Specificity	PPV	IOU	F1
A	initial	0.9340	5.6570	0.8981	0.9953	0.8685	0.7872	0.8831
	1	0.9005	8.5498	0.9074	0.9892	0.7638	0.7060	0.8294
	2	0.9040	9.7341	0.9079	0.9905	0.7904	0.7240	0.8451
	3	0.9196	8.0357	0.8951	0.9926	0.8180	0.7420	0.8548
	4	0.9136	7.8488	0.9155	0.9909	0.7823	0.7214	0.8437
	5	0.9089	10.0914	0.9047	0.9921	0.7736	0.7083	0.8340
B	initial	0.9328	5.0626	0.9337	0.9956	0.8196	0.7659	0.8729
	1	0.9115	6.8731	0.9129	0.9950	0.7937	0.7339	0.8492
	2	0.9258	4.8663	0.9196	0.9960	0.8194	0.7525	0.8666
	3	0.9304	5.5225	0.9043	0.9965	0.8305	0.7601	0.8658
	4	0.9170	4.9035	0.9139	0.9950	0.7983	0.7443	0.8522
	5	0.9371	3.4074	0.9290	0.9972	0.8418	0.7845	0.8832
C	initial	0.9620	2.8192	0.9539	0.9973	0.9076	0.8672	0.9302
	1	0.9485	3.8772	0.9271	0.9970	0.8897	0.8270	0.9080
	2	0.9569	3.5448	0.9298	0.9972	0.9013	0.8421	0.9153
	3	0.9543	3.5278	0.9291	0.9969	0.8956	0.8377	0.9120
	4	0.9466	4.6275	0.9424	0.9955	0.8563	0.8100	0.8973
	5	0.9592	2.5650	0.9381	0.9978	0.9246	0.8695	0.9313
D	initial	0.9191	9.4369	0.8434	0.9966	0.8370	0.7465	0.8402
	1	0.9281	7.4399	0.9150	0.9947	0.8320	0.7788	0.8715
	2	0.9255	7.2161	0.9125	0.9956	0.8327	0.7777	0.8708
	3	0.9334	6.8064	0.9198	0.9946	0.8150	0.7627	0.8642
	4	0.9476	6.0605	0.9210	0.9957	0.8426	0.7822	0.8800
	5	0.9243	9.7444	0.9096	0.9943	0.8044	0.7509	0.8538
E	initial	0.9383	6.0202	0.9296	0.9946	0.8501	0.8028	0.8881
	1	0.9131	9.6877	0.9203	0.9913	0.8024	0.7439	0.8573
	2	0.9308	6.9545	0.9291	0.9915	0.8188	0.7703	0.8705
	3	0.9166	9.4326	0.9038	0.9896	0.7892	0.7457	0.8426
	4	0.9159	8.4609	0.9331	0.9900	0.7800	0.7418	0.8497
	5	0.9329	5.1608	0.9026	0.9949	0.8570	0.7879	0.8792
F	initial	0.9080	6.8139	0.8981	0.9942	0.7973	0.7445	0.8447
	1	0.9022	6.5187	0.8782	0.9957	0.8097	0.7134	0.8425
	2	0.8932	7.9375	0.8689	0.9939	0.7801	0.7069	0.8221
	3	0.9083	8.1140	0.8775	0.9956	0.8148	0.7402	0.8450
	4	0.9041	7.3885	0.9216	0.9932	0.7722	0.7246	0.8403
	5	0.8918	7.7375	0.8909	0.9953	0.7952	0.7166	0.8403

Notes: ASSD, Average Symmetric Surface Distance; PPV, Positive Predictive Value; IOU, Intersection Over Union. Source data are provided as a Source Data file.

Supplementary Table 14. Performance of VFMGL under various data distributions in use case 4

Center	Distribution	Dice	ASSD	Sensitivity	Specificity	PPV	IOU	F1
A	Initial	0.7509	3.6429	0.5774	0.9606	0.8257	0.4918	0.6796
	1	0.7574	6.6052	0.6516	0.9404	0.6632	0.4406	0.6573
	2	0.7955	6.4820	0.7688	0.9170	0.6340	0.5054	0.6949
	3	0.7820	6.1335	0.7001	0.9318	0.6701	0.4895	0.6848
	4	0.7715	6.1798	0.7149	0.9286	0.6423	0.4700	0.6766
	5	0.7415	6.7174	0.6362	0.9346	0.6238	0.4323	0.6300
B	Initial	0.7658	4.1422	0.6245	0.9347	0.7240	0.4731	0.6706
	1	0.7717	4.6163	0.6674	0.9366	0.6840	0.4777	0.6756
	2	0.7816	4.7190	0.6996	0.9156	0.6239	0.4653	0.6596
	3	0.7559	7.7385	0.7909	0.8771	0.5099	0.4388	0.6201
	4	0.7528	4.9399	0.6696	0.9221	0.6399	0.4586	0.6545
	5	0.7632	7.0551	0.7379	0.9100	0.5853	0.4341	0.6528
C	Initial	0.7735	8.2582	0.6203	0.9646	0.7653	0.4823	0.6852
	1	0.7700	9.7468	0.7477	0.9253	0.5885	0.4502	0.6586
	2	0.7703	10.6894	0.7357	0.9227	0.6009	0.4593	0.6615
	3	0.7733	10.9627	0.7115	0.9201	0.5707	0.4314	0.6334
	4	0.8176	5.8284	0.7548	0.9307	0.6793	0.5251	0.7151
	5	0.7893	9.0939	0.7107	0.9484	0.6529	0.4861	0.6806
D	Initial	0.7410	3.6780	0.5059	0.9681	0.8440	0.4471	0.6326
	1	0.7793	6.4168	0.7545	0.9068	0.6190	0.4808	0.6801
	2	0.7825	5.6679	0.7487	0.9064	0.6292	0.4945	0.6838
	3	0.7794	4.8177	0.6492	0.9390	0.7212	0.4926	0.6833
	4	0.7647	5.7593	0.6518	0.9286	0.6955	0.4647	0.6729
	5	0.7794	5.2083	0.6974	0.9207	0.6746	0.5014	0.6858
E	Initial	0.7568	1.0707	0.6386	0.8806	0.6821	0.4914	0.6596
	1	0.7552	1.1665	0.6574	0.8775	0.6549	0.4851	0.6562
	2	0.7506	1.1697	0.6480	0.8822	0.6840	0.4938	0.6655
	3	0.7746	1.0311	0.6804	0.8951	0.7071	0.5265	0.6935
	4	0.7492	1.0805	0.6696	0.8875	0.6733	0.4946	0.6714
	5	0.7509	1.2700	0.6420	0.8854	0.6368	0.4557	0.6394
F	Initial	0.7899	4.1967	0.6218	0.9729	0.7339	0.4772	0.6732
	1	0.8276	3.9756	0.7545	0.9645	0.7097	0.5415	0.7314
	2	0.8065	6.1754	0.8185	0.9563	0.6114	0.4968	0.7000
	3	0.8357	3.7802	0.7583	0.9692	0.7242	0.5574	0.7408
	4	0.8446	4.2443	0.7073	0.9753	0.7540	0.5446	0.7299
	5	0.8358	8.2434	0.7934	0.9725	0.6613	0.5383	0.7213

Notes: ASSD, Average Symmetric Surface Distance; PPV, Positive Predictive Value; IOU, Intersection Over Union. Source data are provided as a Source Data file.

Supplementary Table 15. The matching differences of model layers between the local model of different centers and the vision foundation model

Center \ matching	(0, 0)	(0, 1)	(0, 2)	(0, 3)	(0, 4)	(1, 0)	(1, 1)	(1, 2)	(1, 3)	(1, 4)
Center A	0.08	1.2	0.98	4.22	3.33	0.99	3.48	3.04	0.43	0.03
Center B	0.78	2.81	1.89	2.4	0.05	0.	4.55	0.15	0.52	0.78
Center C	2.74	3.71	0.	0.	3.42	2.14	1.47	1.96	1.41	0.
Center D	0.	1.61	0.15	0.17	0.	1.64	1.04	1.45	0.05	0.01

Center \ matching	(2, 0)	(2, 1)	(2, 2)	(2, 3)	(2, 4)	(3, 0)	(3, 1)	(3, 2)	(3, 3)	(3, 4)
Center A	1.47	0.41	0.62	1.22	1.02	0.95	1.65	2.01	0.1	0.38
Center B	0.	2.18	0.	1.91	0.76	0.99	0.22	0.61	0.63	0.85
Center C	2.21	0.81	0.	0.94	0.4	0.01	1.27	1.63	0.42	0.43
Center D	0.47	1.65	1.56	1.81	1.81	1.37	0.82	0.2	1.16	0.9

The table shows that in use case 1, the matching degree between vision foundation model (VFM) model layers and local model layers varies across centers. For example, (0,0) indicates a pre-matching between layer 0 of the VFM and layer 0 of the local model, with the local model in Center C exhibiting a higher degree of adaptation. In the (0,3) matching, Centers A and B demonstrate a higher level of adaptation. Source data are provided as a Source Data file.

Supplementary Table 16. Performance comparison between VFMGL and the KD method based on model logic layer output.

Center	Methods	AUC	Sensitivity	Specificity	Accuracy	PPV	NPV	F1
A	VFMGL	0.9992	0.9882 (5874/5944)	0.9896 (5882/5944)	0.9889 (11756/11888)	0.9896 (5874/5936)	0.9882 (5882/5952)	0.9889
	Virchow_KD	0.9266	0.8612 (5119/5944)	0.7873 (4680/5944)	0.8243 (9799/11888)	0.8020 (5119/6383)	0.8501 (4680/5505)	0.8305
B	VFMGL	0.9973	0.9481 (3309/3490)	0.9974 (3482/3491)	0.9728 (6791/6981)	0.9973 (3309/3318)	0.9506 (3482/3663)	0.9721
	Virchow_KD	0.9981	0.9436 (3293/3490)	0.9946 (3472/3491)	0.9691 (6765/6981)	0.9943 (3293/3312)	0.9463 (3472/3669)	0.9682
C	VFMGL	0.9995	0.9911 (8430/8506)	0.9915 (8433/8505)	0.9913 (16863/17011)	0.9915 (8430/8502)	0.9911 (8433/8509)	0.9913
	Virchow_KD	0.6401	0.1743 (1483/8506)	0.9949 (8462/8505)	0.5846 (9945/17011)	0.9718 (1483/1526)	0.5465 (8462/15485)	0.2957
D	VFMGL	0.9977	0.9427 (12240/12984)	0.9988 (12969/12984)	0.9708 (25209/25968)	0.9988 (12240/12255)	0.9457 (12969/13713)	0.9699
	Virchow_KD	0.9748	0.7160 (9297/12984)	0.9970 (12945/12984)	0.8565 (22242/25968)	0.9958 (9297/9336)	0.7783 (12945/16632)	0.8331
E	VFMGL	0.9993	0.9845 (14445/14672)	0.9922 (14559/14673)	0.9884 (29004/29345)	0.9922 (14445/14559)	0.9846 (14559/14786)	0.9883
	Virchow_KD	0.9157	0.1322 (1940/14672)	0.9957 (14610/14673)	0.5640 (16550/29345)	0.9685 (1940/2003)	0.5343 (14610/27342)	0.2327

Notes: VFMGL, Vision Foundation Model General Lightweight; KD, Knowledge Distillation; AUC, Area Under the Curve; PPV, Positive Predictive Value; NPV, Negative Predictive Value. Source data are provided as a Source Data file.

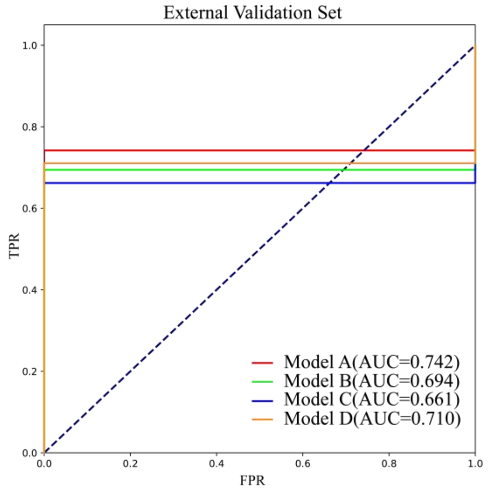

Supplementary Fig.1 The ROC curves for VFMGL on external center E is shown. Notes: ROC, Receiver Operating Characteristic curve; AUC, Area Under the Curve; TPR, True Positive Rate; FPR, False Positive Rate. Source data are provided as a Source Data file.

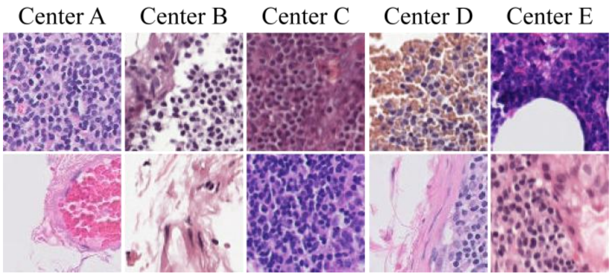

Supplementary Fig.2 Examples of breast histology images of normal and tumor tissues from five centers.

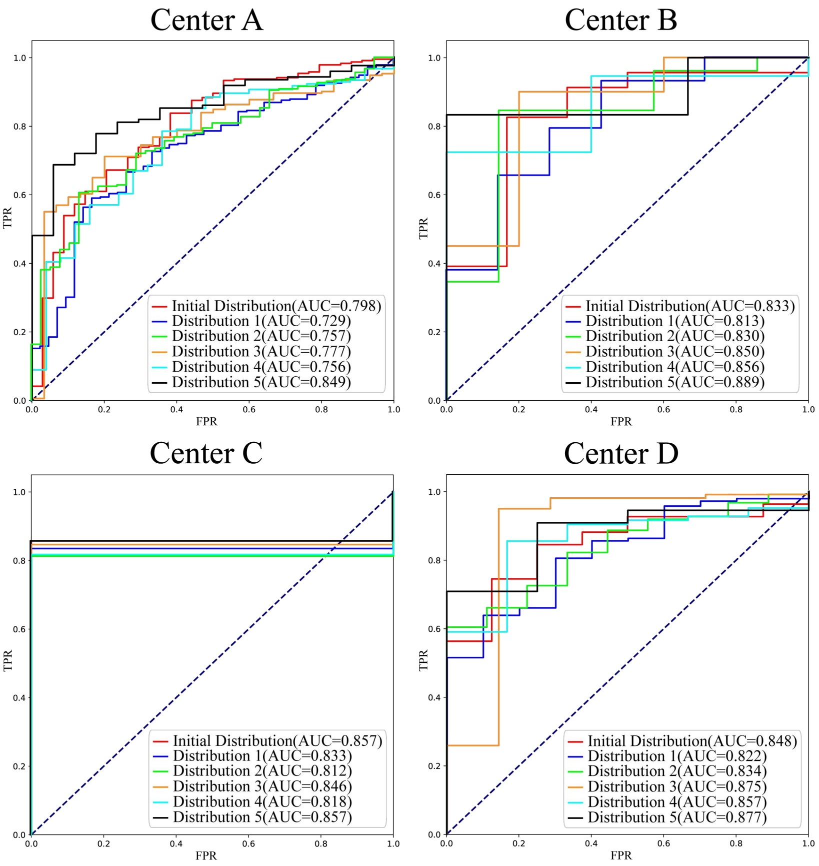

Supplementary Fig.3 ROC curves of VFMGL under different data distributions at the four centers. Notes: VFMGL, Vision Foundation Model General Lightweight; ROC, Receiver Operating Characteristic curve; AUC, Area Under the Curve; TPR, True Positive Rate; FPR, False Positive Rate. Source data are provided as a Source Data file.

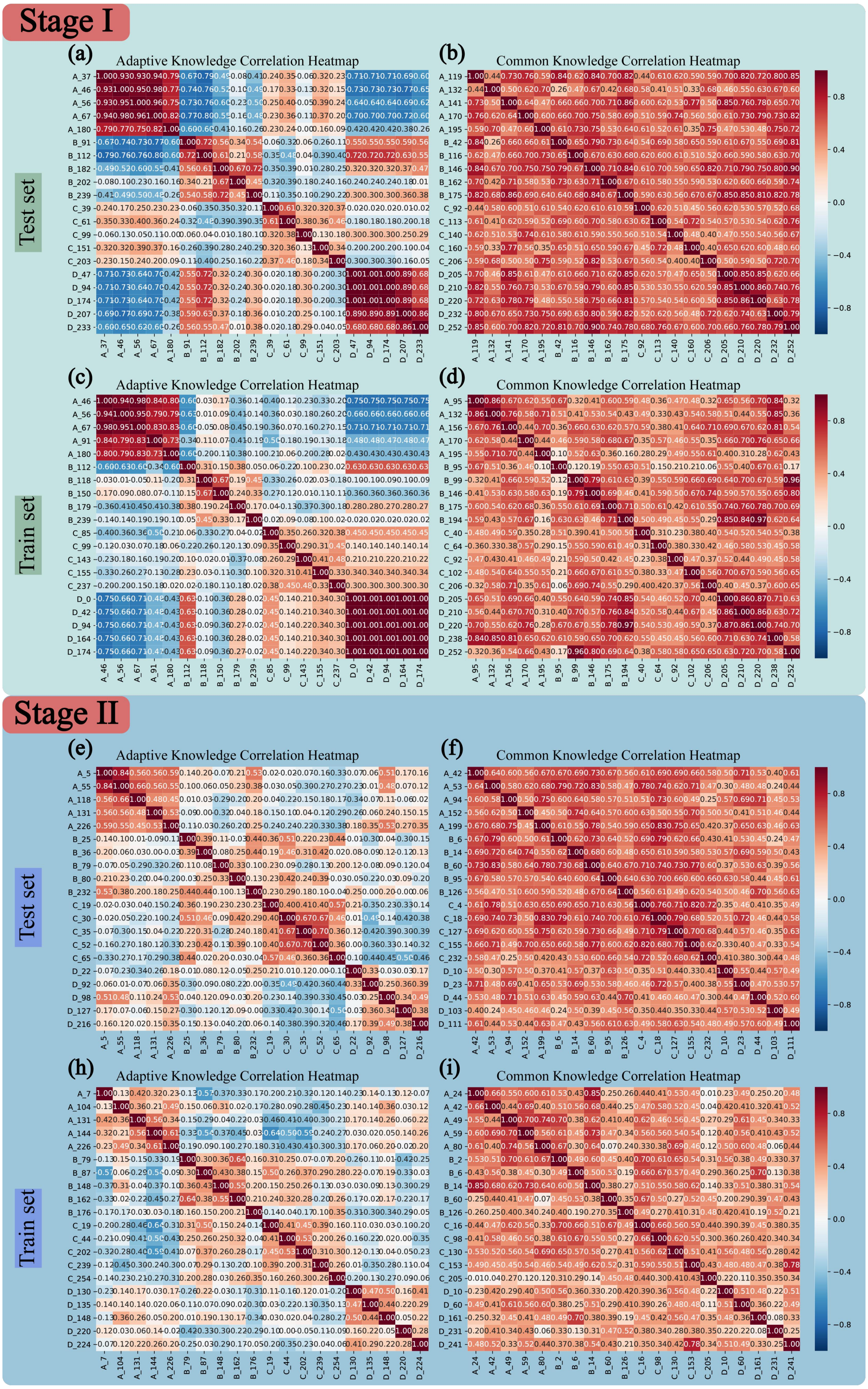

Supplementary Fig.4 Correlation heatmaps of adaptive knowledge and common knowledge. (a) Adaptive knowledge correlation heatmap of the first stage of VFMGL in the test set. **(b)** Common knowledge correlation heatmap of the first stage of VFMGL in the test

set. **(c)** Adaptive knowledge correlation heatmap of the first stage of VFMGL in the training set. **(d)** Common knowledge correlation heatmap of the first stage of VFMGL in the training set. **(e)** Adaptive knowledge correlation heatmap of the second stage of VFMGL in the test set. **(f)** Common knowledge correlation heatmap of the second stage of VFMGL in the test set. **(g)** Adaptive knowledge correlation heatmap of the second stage of VFMGL in the training set. **(h)** Common knowledge correlation heatmap of the second stage of VFMGL in the training set. The first column shows the heatmap of correlation for adaptive features, while the second column displays the heatmap of correlation for common features. Notes: VFMGL, Vision Foundation Model General Lightweight. Source data are provided as a Source Data file.

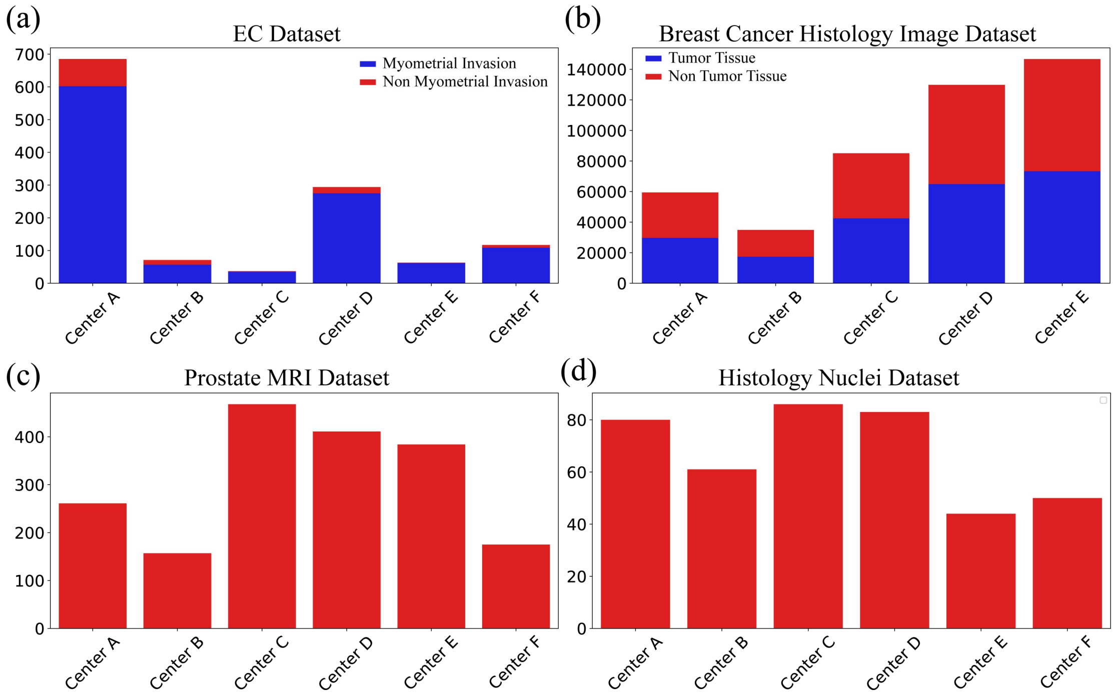

Supplementary Fig.5 Data distributions of use case 1-4. (a) The multi-center distributions for use cases 1. **(b)** The multi-center distributions for use cases 2. **(c)** The multi-center distributions for use cases 3. **(d)** The multi-center distributions for use cases 4. Source data are provided as a Source Data file.

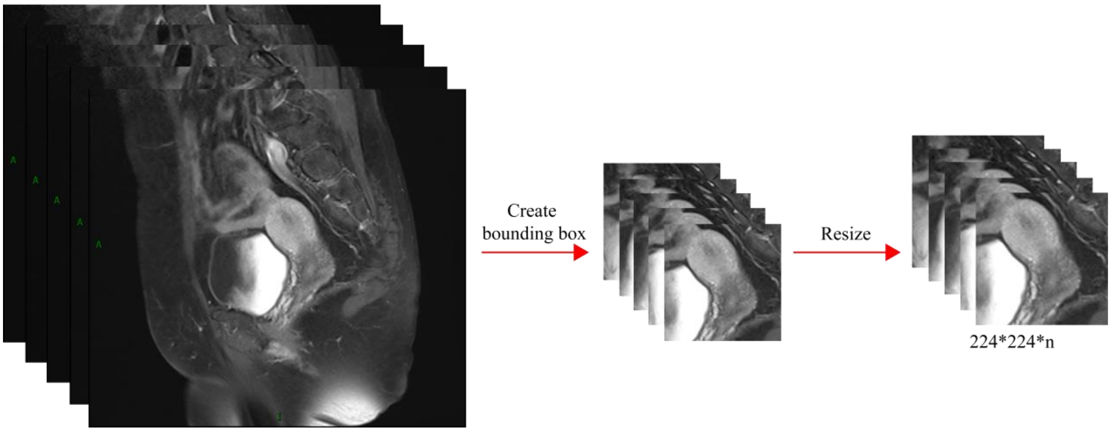

Supplementary Fig.6 The preprocessing of CT images for a single patient is shown above.
Notes: n is the number of CT images the patient has; CT, Computed Tomography.

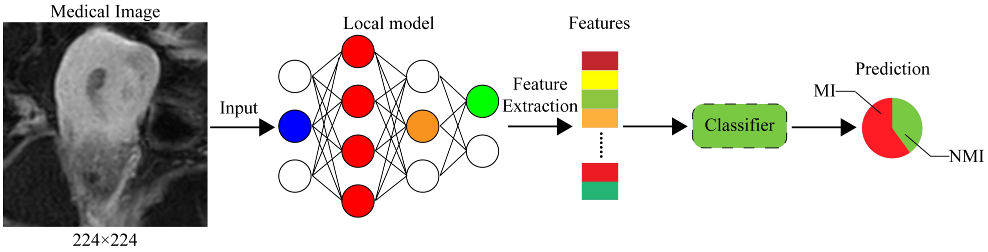

Supplementary Fig.7 Feature extraction and patient classification. Notes: MI, Myometrial Invasion; NMI, Non Myometrial Invasion.

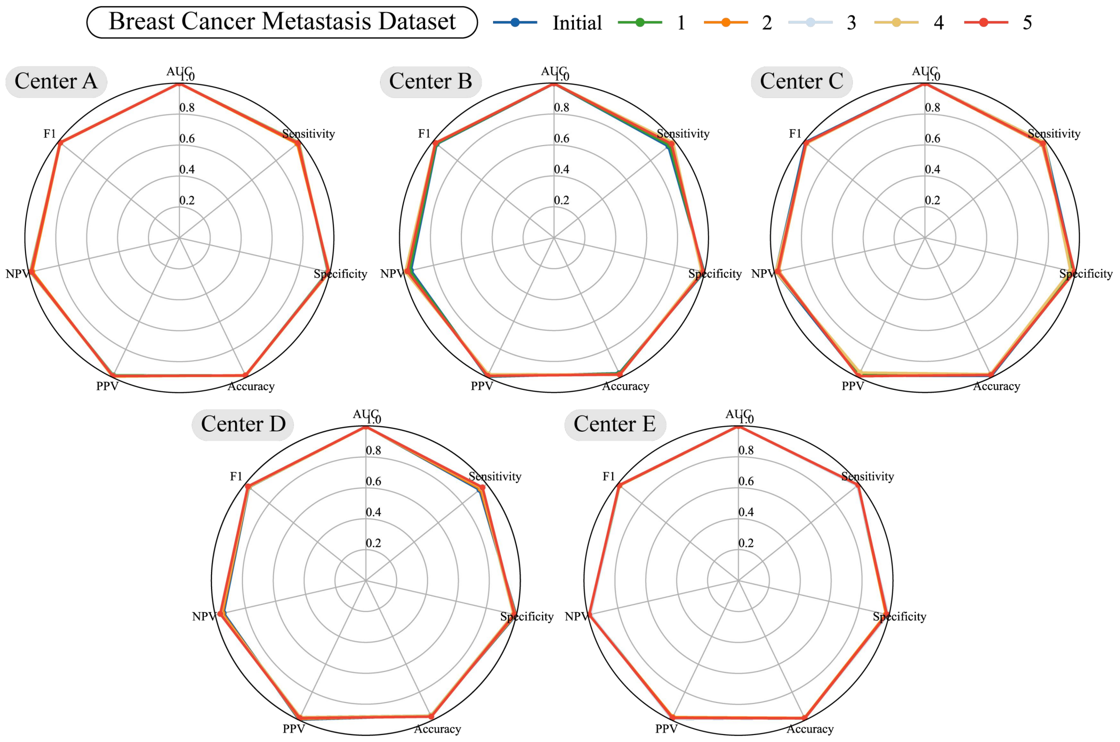

Supplementary Fig.8 Radar chart comparison of VFMGL's performance under six different data distributions in the breast cancer histology image classification task(use case 2). Notes: AUC, Area Under the Curve; PPV, Positive Predictive Value; NPV, Negative Predictive Value; VFMGL, Vision Foundation Model General Lightweight. Source data are provided as a Source Data file.

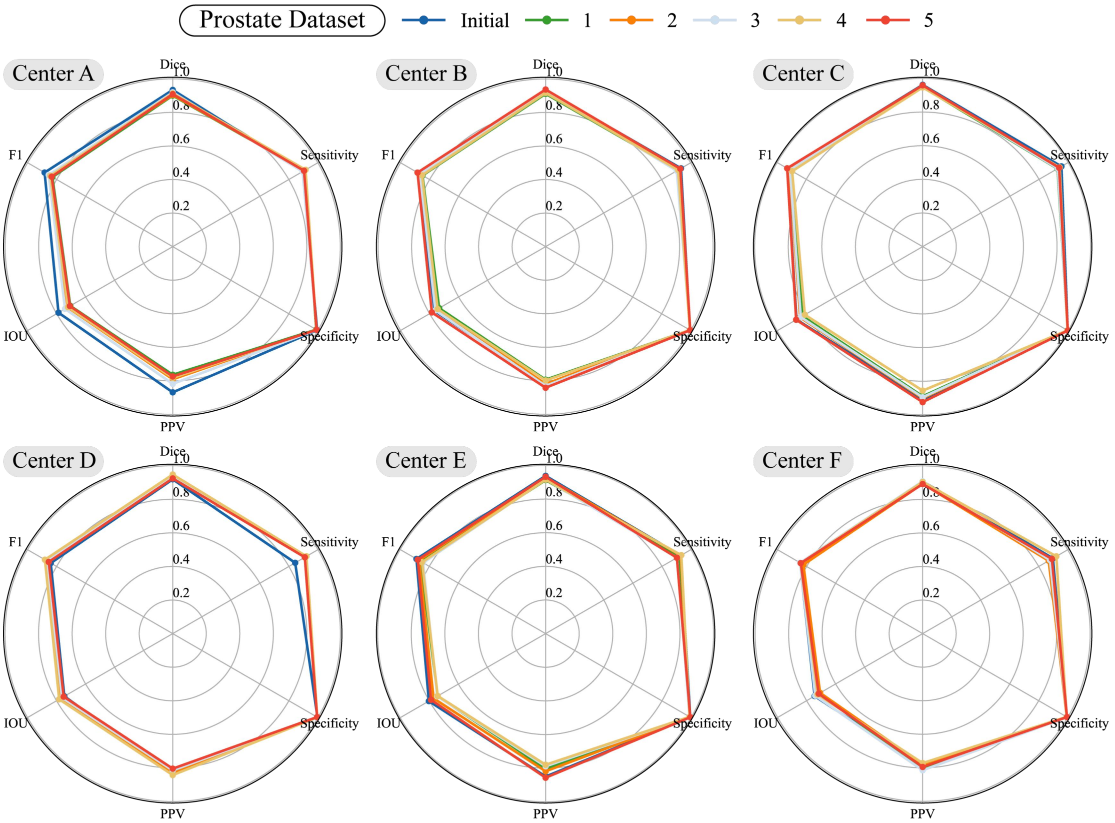

Supplementary Fig.9 Radar chart comparison of VFMGL's performance under six different data distributions in the prostate MRI segmentation task(use case 3). Notes: PPV, Positive Predictive Value; IOU, Intersection Over Union; VFMGL, Vision Foundation Model General Lightweight; MRI, Magnetic Resonance Imaging. Source data are provided as a Source Data file.

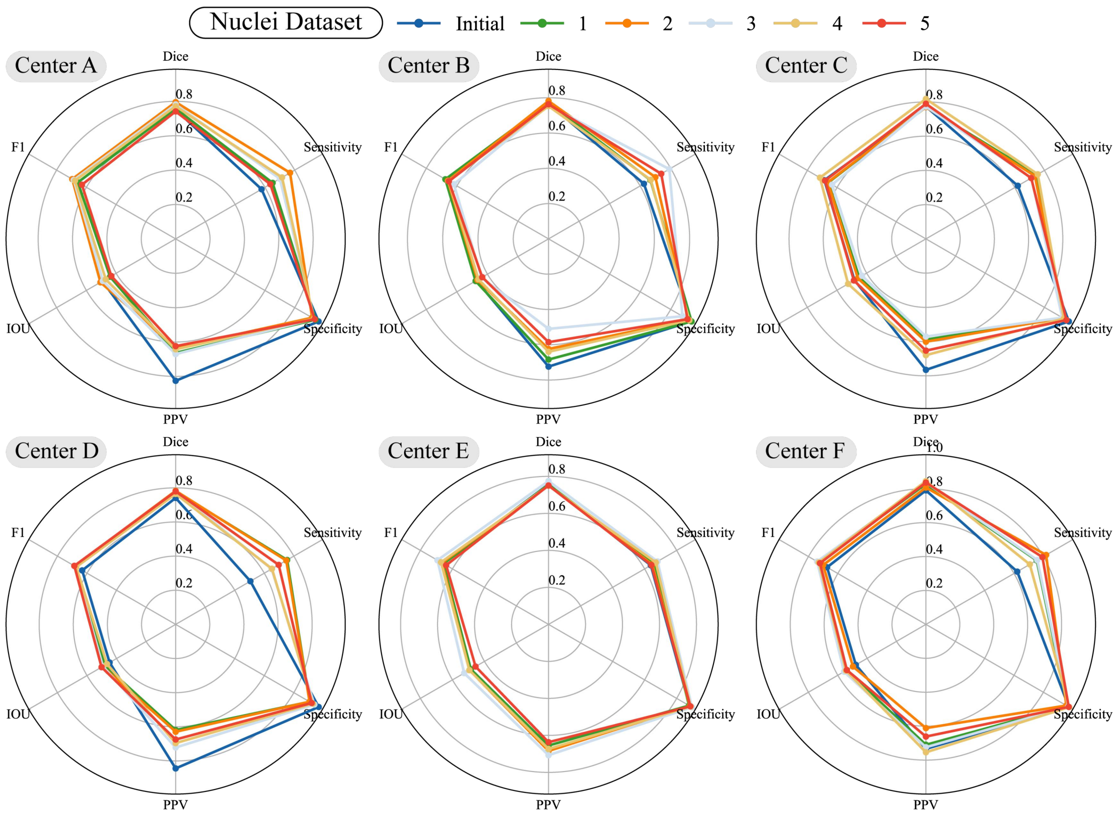

Supplementary Fig.10 Radar chart comparison of VFMGL's performance under six different data distributions in the histology nuclei segmentation task(use case 4). Notes: PPV, Positive Predictive Value; IOU: Intersection Over Union; VFMGL, Vision Foundation Model General Lightweight. Source data are provided as a Source Data file.

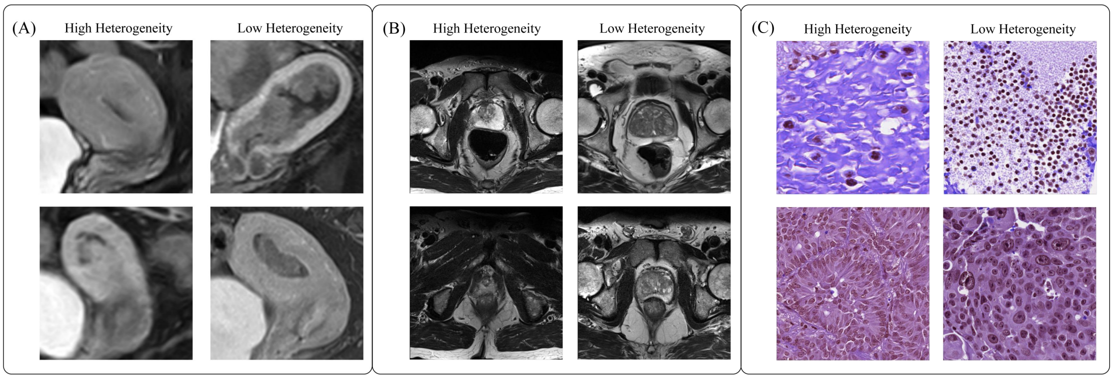

Supplementary Fig.11 Examples of high heterogeneity and low heterogeneity. (A) Display of high and low heterogeneity data in use case 1. **(B)** Display of high and low heterogeneity data in use case 3. **(C)** Display of high and low heterogeneity data in use case 4. As shown in Supplementary Fig.11(A), in the example provided for use case 1, the first column represents examples identified by DDBL as high-heterogeneity data, while the second column shows low-heterogeneity data; the first row contains positive samples, and the second row, negative samples. The figure demonstrates that low-heterogeneity data provide simpler and clearer information useful for diagnosis, with a lower risk of misjudgment. In contrast, high-heterogeneity data offer more complex information, which increases diagnostic difficulty and risk—such as how the intrauterine environment and the smoothness of the inner wall can impact diagnosis. In segmentation tasks (Supplementary Fig.11(B)(C)), the segmentation targets in low-heterogeneity images exhibit relatively clear and common boundary information, whereas high-heterogeneity images present segmentation targets with more challenging contours to define, for example, the impact of staining differences on the delineation of nuclear contours.

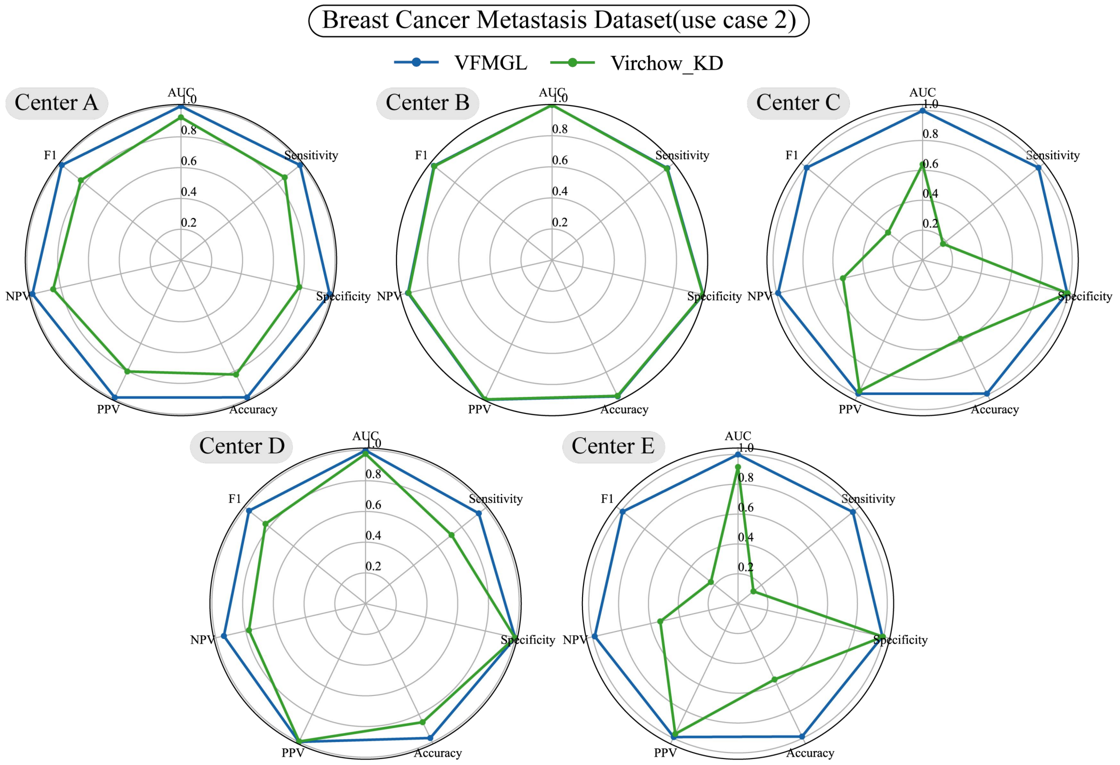

Supplementary Fig.12 Performance comparison between VFMGL and Logit-based Knowledge Distillation in use case 2. Notes: AUC, Area Under the Curve; PPV, Positive Predictive Value; NPV, Negative Predictive Value; VFMGL, Vision Foundation Model General Lightweight; KD, Knowledge Distillation. Logit-based Knowledge Distillation (KD) helps the student model achieve better generalization in classification tasks by aligning its probability distribution predictions with those of the teacher model, usually requiring the student and teacher models to share the same label space. To compare the performance of VFMGL with the logit-based KD method on multi-center medical data, we used Virchow and ResNet18 as the teacher and student models, respectively, for the logit-based KD method. The Virchow model, based on the DINOv2 framework, was pre-trained on 1.5 million whole-slide histopathology images (including breast cancer pathology images) and then used model embeddings and nearly 80,000 annotated medical samples to build a classifier for specific downstream tasks. Therefore, in the breast cancer pathology image classification task (use case 2), we compared VFMGL with the logit-based KD method (Fig. S12). The results indicate that VFMGL achieved higher scores across multiple metrics on multi-center data, with stable predictive performance. Further details of the results are provided in Supplementary Table 16. Source data are provided as a Source Data file.

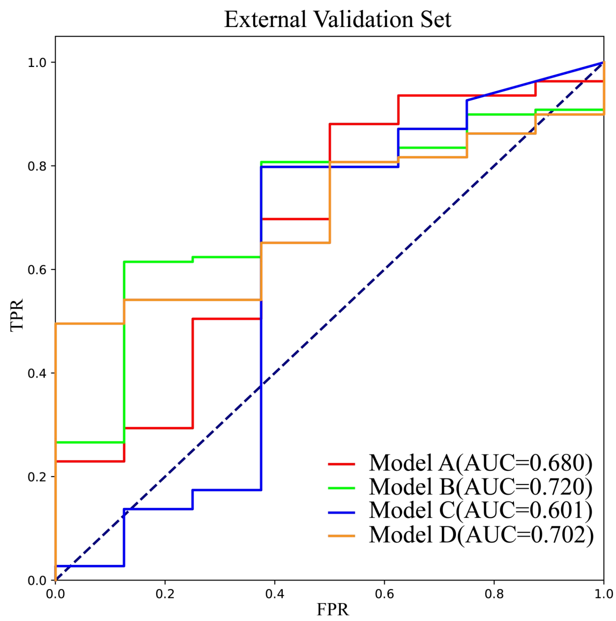

Supplementary Fig.13 The ROC curves for VFMGL on external center F is shown. Notes: ROC, Receiver Operating Characteristic curve; AUC, Area Under the Curve; TPR, True Positive Rate; FPR, False Positive Rate. Source data are provided as a Source Data file.

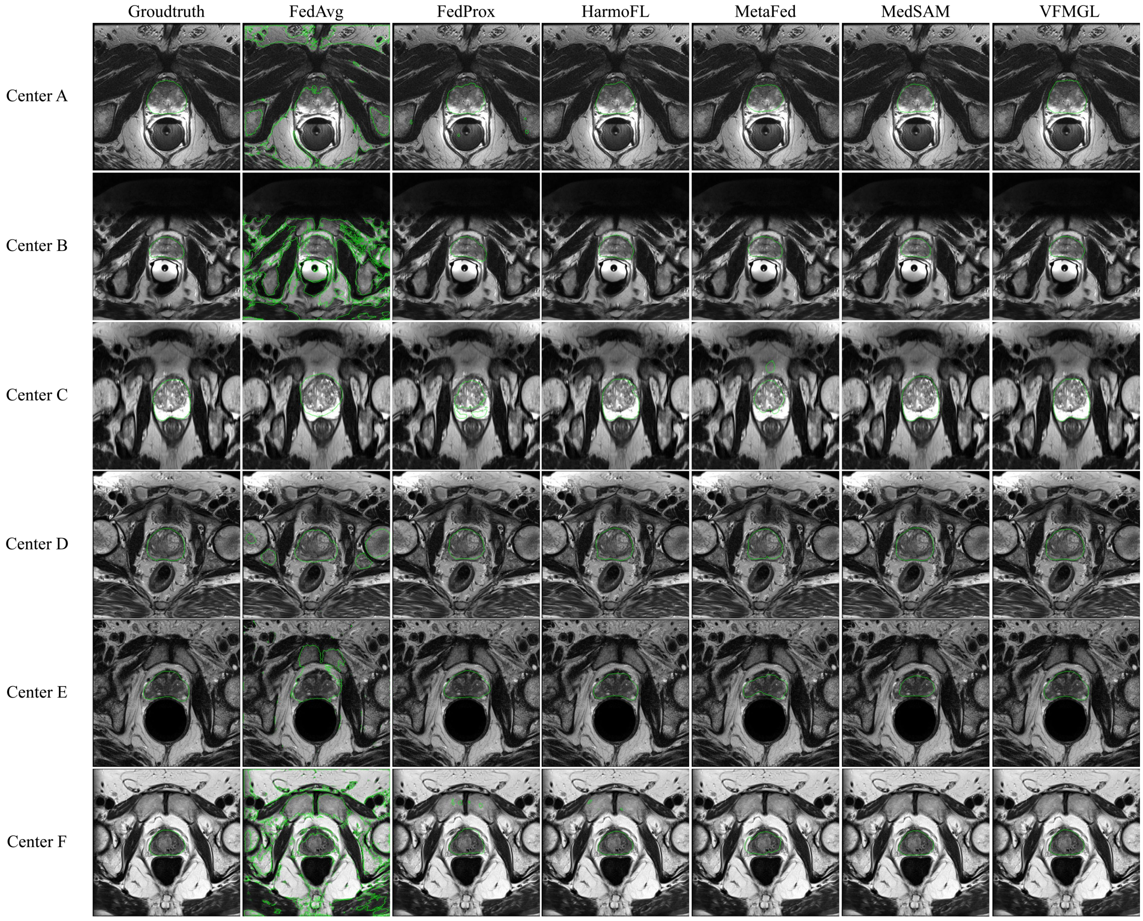

Supplementary Fig.14 The segmentation results of the prostate along with comparisons. Rows 1-6 depict examples from Centers A-F respectively, where the first column represents the ground truth labels, and columns 2-7 represent the segmentation results of each method. Notes: VFMGL, Vision Foundation Model General Lightweight.

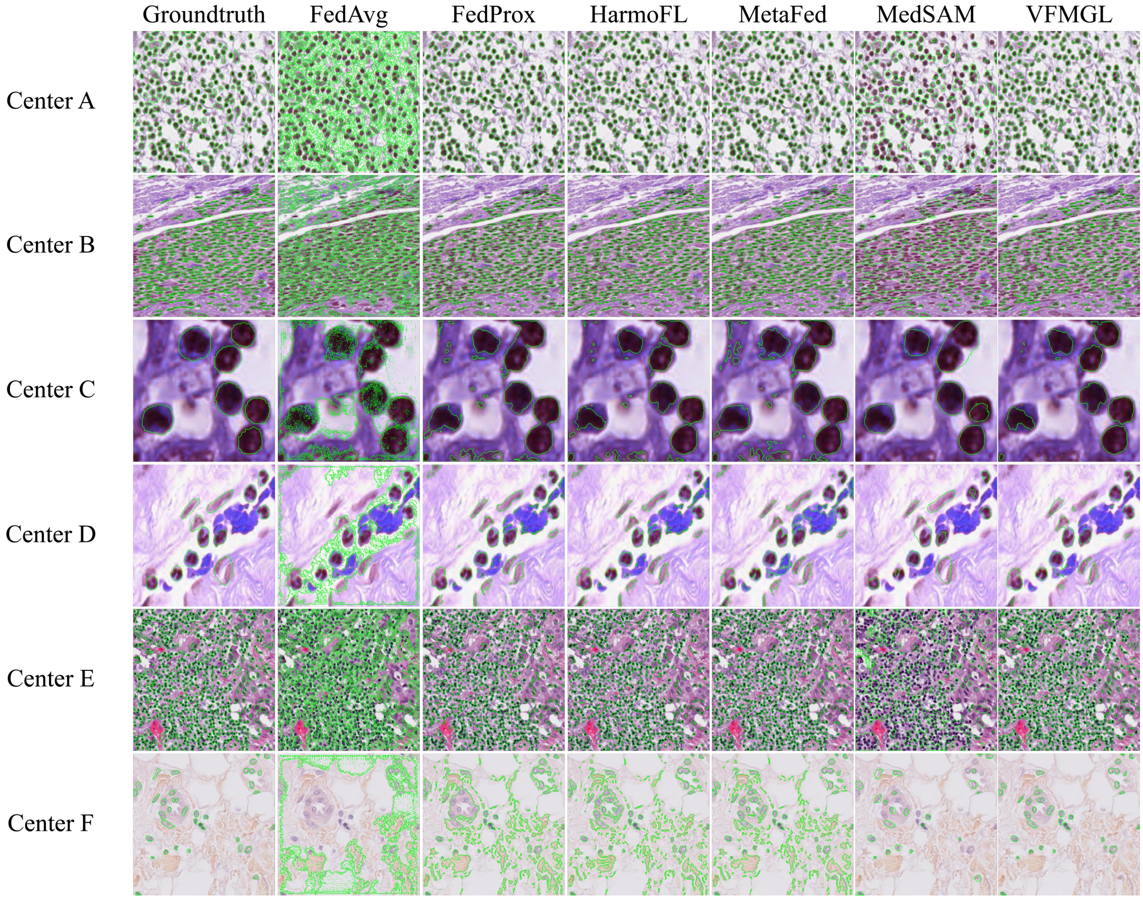

Supplementary Fig.15 The segmentation results of the cell nuclei along with comparisons. Rows 1-6 depict examples from Centers A-F respectively, where the first column represents the ground truth labels, and columns 2-7 represent the segmentation results of each method. Notes: VFMGL, Vision Foundation Model General Lightweight.

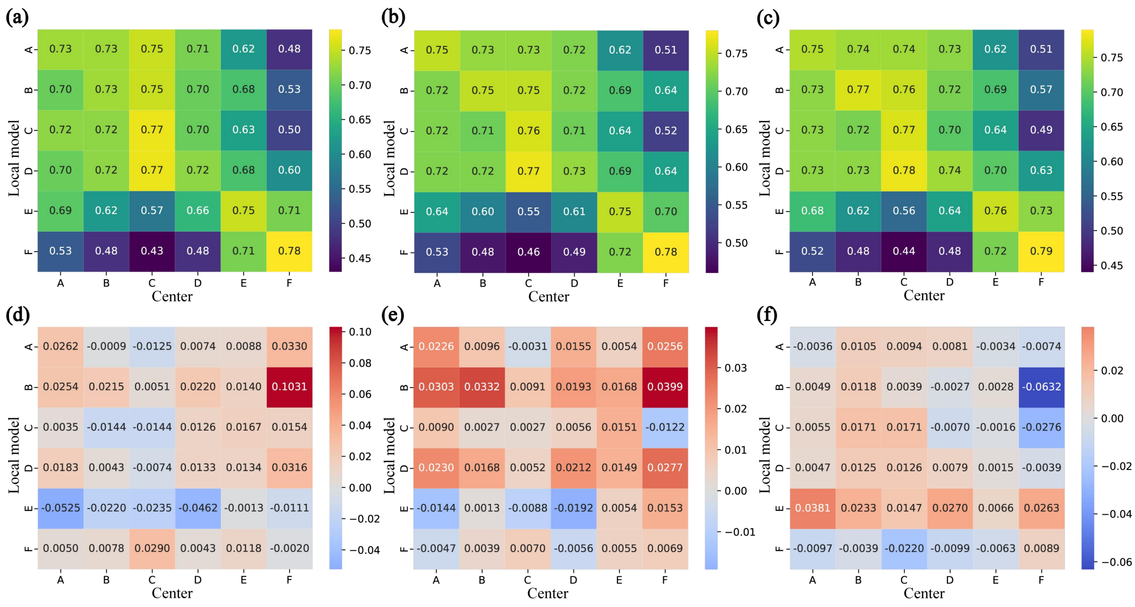

Supplementary Fig.16 Ablation experiments of VFMGL in use case 4. (a) Performance when constructing models using only the HGKT method. **(b)** Performance when constructing models using only the HGKT+KD method. **(c)** Performance when constructing models using the HGKT+KD+DDBL method. **(d)** Models performance change from subfigure (a) to (b). **(e)** Models performance change from subfigure (a) to (c). **(f)** Models performance change from subfigure (b) to (c). Notes: HGKT, Heterogeneous-model General Knowledge Transfer; KD, Knowledge Distillation; DDBL, Data Deduction in Batch Level. Source data are provided as a Source Data file.

Supplementary References

1. Luo, J., Vong, C.-M. & Wong, P.-K. Sparse Bayesian extreme learning machine for multi-classification. IEEE Trans. Neural Netw. Learn Syst. **25**, 836–843 (2014).
