# Supplementary material for: General lightweight framework for vision foundation model supporting multi-task and multi-center medical image analysis
Source: Nat Commun. 2025 Mar 1;16:2097. doi: 10.1038/s41467-025-57427-z (PMC11873151; doi:10.1038/s41467-025-57427-z)
Supplement: Supplementary file 2 — Reporting Summary [file 41467_2025_57427_MOESM2_ESM.pdf]

nature portfolio

Corresponding author(s): Bao Feng, Wansheng Long

Last updated by author(s): Feb 13, 2025

Reporting Summary

Nature Portfolio wishes to improve the reproducibility of the work that we publish. This form provides structure for consistency and transparency in reporting. For further information on Nature Portfolio policies, see our Editorial Policies and the Editorial Policy Checklist.

Statistics

For all statistical analyses, confirm that the following items are present in the figure legend, table legend, main text, or Methods section.

n/a	Confirmed	
☐	☒	The exact sample size (*n*) for each experimental group/condition, given as a discrete number and unit of measurement
☐	☒	A statement on whether measurements were taken from distinct samples or whether the same sample was measured repeatedly
☐	☒	The statistical test(s) used AND whether they are one- or two-sided<br>*Only common tests should be described solely by name; describe more complex techniques in the Methods section.*
☒	☐	A description of all covariates tested
☒	☐	A description of any assumptions or corrections, such as tests of normality and adjustment for multiple comparisons
☐	☒	A full description of the statistical parameters including central tendency (e.g. means) or other basic estimates (e.g. regression coefficient) AND variation (e.g. standard deviation) or associated estimates of uncertainty (e.g. confidence intervals)
☐	☒	For null hypothesis testing, the test statistic (e.g. *F*, *t*, *r*) with confidence intervals, effect sizes, degrees of freedom and *P* value noted<br>*Give P values as exact values whenever suitable.*
☒	☐	For Bayesian analysis, information on the choice of priors and Markov chain Monte Carlo settings
☐	☒	For hierarchical and complex designs, identification of the appropriate level for tests and full reporting of outcomes
☐	☒	Estimates of effect sizes (e.g. Cohen's *d*, Pearson's *r*), indicating how they were calculated

Our web collection on statistics for biologists contains articles on many of the points above.

Software and code

Policy information about availability of computer code

Data collection	No software was used
Data analysis	Python(version 3.9.18) was used to complete the deep learning framework; Pytorch(version 2.0.0+cu117) was used to build the general lightweight framework for vision foundation model; Matlab(version 2021b) was used for feature screening; statistical tests were performed using SPSS(SPSS Statistics 26.0); The codes for the method proposed in this paper are made publicly available on GitHub:https://github.com/baofengguat/VFMGL/tree/main

For manuscripts utilizing custom algorithms or software that are central to the research but not yet described in published literature, software must be made available to editors and reviewers. We strongly encourage code deposition in a community repository (e.g. GitHub). See the Nature Portfolio guidelines for submitting code & software for further information.

Data

Policy information about availability of data

All manuscripts must include a data availability statement. This statement should provide the following information, where applicable:

- Accession codes, unique identifiers, or web links for publicly available datasets
- A description of any restrictions on data availability
- For clinical datasets or third party data, please ensure that the statement adheres to our policy

The EC dataset (use case1) in the current study are not publicly available for patient privacy policy. However, if researchers wish to access our data solely for scientific research purposes, access can be obtained by sending an email request to the corresponding author. Requests will be processed by the corresponding author within 3 months and followed up with the requesting party. Any requests will be pending prior approval and revision by the Ethics Committee of Jiangmen Central Hospital, the Ethics Committee of Yuebei People's Hospital, the Ethics Committee of Affiliated Dongguan Hospital Southern Medical University, the Ethics Committee of Maoming People's Hospital, the Ethics Committee of Kaiping Central Hospital and the Ethics Committee of the Third Affiliated Hospital of Guangzhou Medical University, which retain all rights to deny access. The Breast Cancer Histology Image dataset (use case 2) used in this study are available in link: https://worksheets.codalab.org/rest/bundles/0xe45e15f39fb54e9d9e919556af67aabe/contents/blob/. The Prostate MRI dataset (use case 3) used in this study are available in link: https://liuquande.github.io/SAML/. The Histology Nuclei dataset (use case 4) used in this study are available in link: https://monusac-2020.grand-challenge.org/Data/; https://zenodo.org/record/1175282/files/TNBC_NucleiSegmentation.zip; https://monuseg.grand-challenge.org/Data/. The deidentified relevant data generated in this study are provided in the Supplementary Information/Source Data file and can be downloaded from the following link: https://pan.baidu.com/s/1ZOzXlsG3ez3F9xyxsKZD8g?pwd=cyww, with the access code: cyww.Source data are provided with this paper.

Research involving human participants, their data, or biological material

Policy information about studies with human participants or human data. See also policy information about sex, gender (identity/presentation), and sexual orientation and race, ethnicity and racism.

Reporting on sex and gender	The gender mentioned in the data used in this study refers to biological sex.
Reporting on race, ethnicity, or other socially relevant groupings	This study does not involve reporting on race, ethnicity, or other socially relevant groupings.
Population characteristics	EC dataset: A total of 1,267 patients from six hospitals were included in this study. The average age of patients in Center A was 54.7 years; 91.7% (n=628) were for Histopathologic Type I and 8.3% (n=57) were for Type II. The distribution of Histopathologic Grades was 25.3% (n=173) for Grade 1, 52.4% (n=359) for Grade 2, and 22.3% (n=153) for Grade 3. In Center B, the average age was 52.6 years; 95.8% (n=68) of patients were for Histopathologic Type I and 4.2% (n=3) were for Type II. The Histopathologic Grades were 57.7% (n=41) for Grade 1, 28.2% (n=20) for Grade 2, and 14.1% (n=10) for Grade 3. In Center C, the average age was 60.7 years; 91.9% (n=34) were for Histopathologic Type I and 8.1% (n=3) were for Type II. The Grades were 18.9% (n=7) for Grade 1, 62.2% (n=23) for Grade 2, and 18.9% (n=7) for Grade 3. In Center D, the average age was 54.4 years; 90.8% (n=267) were for Histopathologic Type I and 9.2% (n=27) were for Type II. The distribution of Histopathologic Grades was 48% (n=141) for Grade 1, 34% (n=100) for Grade 2, and 18% (n=53) for Grade 3. In Center E, the average age was 55.1 years; 96.8% (n=61) were for Histopathologic Type I and 3.2% (n=2) were for Type II. The Histopathologic Grades were 17.5% (n=11) for Grade 1, 61.9% (n=39) for Grade 2, and 20.6% (n=13) for Grade 3. In Center F, the average age was 54.9 years; 90.6% (n=106) were for Histopathologic Type I and 9.4% (n=11) were for Type II.
Recruitment	This study falls under the category of retrospective research.
Ethics oversight	This study was implemented under the approval of the Jiangmen Central Hospital, the Yuebei People's Hospital, Affiliated Dongguan Hospital Southern Medical University, the Maoming People's Hospital, the Kaiping Central Hospital and the Third Affiliated Hospital of Guangzhou Medical University.This study obtained approval from the Institutional Review Board. Given its retrospective nature, informed consent was waived.

Note that full information on the approval of the study protocol must also be provided in the manuscript.

Field-specific reporting

Please select the one below that is the best fit for your research. If you are not sure, read the appropriate sections before making your selection.

☒ Life sciences ☐ Behavioural & social sciences ☐ Ecological, evolutionary & environmental sciences

For a reference copy of the document with all sections, see nature.com/documents/nr-reporting-summary-flat.pdf

Life sciences study design

All studies must disclose on these points even when the disclosure is negative.

Sample size	In the EC dataset (use case 1), a total of data from 6 medical centers comprising 1,267 patients were included. The inclusion criteria were: 1) histologically confirmed endometrial cancer (malignant epithelial tumors of the uterus); 2) underwent total hysterectomy; 3) had pelvic MRI images within 21 days before surgery; 4) had complete postoperative pathological results. The exclusion criteria were: 1) the interval between the pelvic MRI examination date and the surgery date exceeded 21 days; 2) received neoadjuvant therapy before surgery; 3) presence of artifacts or poor image quality in the pelvic MRI; 4) concomitant presence of other malignant tumors, such as ovarian cancer, cervical cancer,

	etc. We did not use statistical models to pre-determine the sample size, but our sample size is the same as or larger than that of studies with similar objectives in previous research reports, such as BC. For example, Yan et al. (2022) collected data from 236 patients for a related study on myometrial invasion in endometrial cancer [1]. In addition, the Breast Cancer Histology Image dataset (use case 2), the Histology Nuclei dataset (use case 3), and the Prostate MRI dataset (use case 4) are publicly available datasets. For each medical center, 20% of the data is allocated to the test set, while the remaining 80% is divided into a 4:1 ratio for the training and validation sets.<br>[1]Yan BC, Ma FH, Li Y, Fan YF, et al. An MRI radiomics nomogram improves the accuracy in identifying eligible candidates for fertility-preserving treatment in endometrioid adenocarcinoma. Am J Cancer Res. 12(3), 1056-1068 (2022).
Data exclusions	EC dataset: 1) interval between pelvic MRI examination date and surgery date exceeding 21 days; 2) received neoadjuvant therapy before surgery; 3) presence of artifacts or poor image quality in pelvic MRI; 4) concomitant presence of other malignant tumors, such as ovarian cancer, cervical cancer.
Replication	In this study, we conducted robustness experiments across four datasets, totaling 25 times. Cross-center generalization experiments were carried out 4 times, and ablation experiments were performed 2 times. Comparison experiments with other methods were conducted 25 times.The experiments in this study are reproducible and can yield similar results.
Randomization	The division of the training and testing sets for the multi-center EC dataset in the study employed a random splitting strategy. We used random splitting to divide the training and test sets, and the splitting ratio and random seed have been described in the methods section of the main manuscript and in Supplementary Tables 6, 9-11.
Blinding	The deep learning models were developed on the training cohort. All training was done without any information from the test cohort and the reader study cohort.

Reporting for specific materials, systems and methods

We require information from authors about some types of materials, experimental systems and methods used in many studies. Here, indicate whether each material, system or method listed is relevant to your study. If you are not sure if a list item applies to your research, read the appropriate section before selecting a response.

Materials & experimental systems

n/a	Involved in the study
☒	☐ Antibodies
☒	☐ Eukaryotic cell lines
☒	☐ Palaeontology and archaeology
☒	☐ Animals and other organisms
☒	☐ Clinical data
☒	☐ Dual use research of concern
☒	☐ Plants

Methods

n/a	Involved in the study
☒	☐ ChIP-seq
☒	☐ Flow cytometry
☒	☐ MRI-based neuroimaging

Plants

Seed stocks	This study does not involve this aspect.
Novel plant genotypes	This study does not involve this aspect.
Authentication	This study does not involve this aspect.
